# Systematic Notchplasty in Primary ACL Reconstruction Using Hamstring Autografts: A Prospective Cohort Study on the Rate of Secondary Arthrolysis

**DOI:** 10.3390/jcm14207285

**Published:** 2025-10-15

**Authors:** Adrien Saint-Etienne, Alexandre Hardy, Antonio Miele, Nicolas Lefevre, Olivier Grimaud, Alain Meyer, Yoann Bohu

**Affiliations:** 1 Clinique du Sport Paris, 36 Boulevard Saint Marcel, 75005 Paris, France; alexandre.hardy@me.com (A.H.); antoniomiele86@gmail.com (A.M.); dr.lefevre@chirurgiedusport.com (N.L.); dr.grimaud@chirurgiedusport.com (O.G.); dr.meyer@chirurgiedusport.com (A.M.); dr.bohu@chirurgiedusport.com (Y.B.); 2 CHU Henri Mondor, 51 Avenue du Maréchal de Lattre de Tassigny, 94010 Créteil, France

**Keywords:** ACL reconstruction, hamstring autograft, notchplasty, arthrolysis, arthrofibrosis, impingement

## Abstract

**Background:** Arthrofibrosis requiring arthrolysis is a relevant complication after anterior cruciate ligament (ACL) reconstruction. It has been suggested that intercondylar notch impingement may contribute to this outcome. The aim of this study was to evaluate whether systematic notchplasty during primary ACL reconstruction with hamstring autografts reduces the rate of secondary arthrolysis. **Methods:** Two groups of patients undergoing primary ACL reconstruction were compared: 149 patients without notchplasty and 140 patients with notchplasty, each with a minimum follow-up of 2 years. The incidence of arthrolysis and other complications, functional outcome scores, and return-to-sport data were analyzed. **Results:** No significant difference was observed in the rate of arthrolysis: Seven patients (4.7%) were in the non-notchplasty group, with seven patients (5.0%) in the notchplasty group (*p* = n.s.). Functional outcomes were comparable between groups, with mean subjective IKDC of 86.5, KOOS of 87.5, and Lysholm of 90.9. Return-to-sport rates were similar, and over 90% of patients in both groups reported being satisfied or very satisfied with their outcome. **Conclusions:** Systematic notchplasty during primary ACL reconstruction with hamstring autografts did not reduce the rate of secondary arthrolysis in this underpowered cohort. Arthrofibrosis is multifactorial, and larger studies are needed to clarify whether notchplasty has an independent effect.

## 1. Introduction

One of the most frequent complications following anterior cruciate ligament (ACL) reconstruction is a knee extension deficit during the early postoperative period. This limitation may persist for more than 3 months and become chronic [1], with reported rates ranging from 1% to 10.9% depending on the series [2,3,4,5].

Arthrofibrosis, defined as pathological scar tissue formation leading to stiffness and loss of motion, is a complex and multifactorial complication. Among the potential causes of extension loss, intercondylar notch impingement has been suggested as one possible mechanism, although others—such as graft malposition, overtensioning, or extra-articular procedures—are also implicated.

Intercondylar notch impingement occurs when there is an imbalance between the “container” and the “content”: malposition of the tunnels causing graft–femur conflict [6,7], an oversized graft [7], or a congenitally small and narrow notch [8,9].

Notchplasty, a technically simple procedure performed with a motorized shaver or burr, aims to enlarge the intercondylar space to prevent or treat graft impingement. However, some cadaveric studies have reported that notchplasty alters knee biomechanics by increasing tibial translation and graft stresses [10], potentially impairing graft integration [11] and functional outcomes.

Clinical evidence on notchplasty remains limited. Only two clinical studies have specifically evaluated its effect on extension loss and the risk of arthrolysis [12], highlighting the scarcity of data in this under-reported area.

The present study investigated whether systematic notchplasty during primary single-bundle ACL reconstruction using hamstring autografts reduces the rate of secondary arthrolysis. The primary objective was to compare the incidence of arthrolysis between ACL reconstruction alone and ACL reconstruction with notchplasty. Secondary objectives were to compare complications, functional outcomes, and retear rates between the two groups. The rationale for including notchplasty was based on the hypothesis that enlarging the intercondylar notch might reduce graft impingement and subsequent extension deficits.

## 2. Materials and Methods

A single-center prospective cohort study was initiated in 2012, including all patients operated on by five senior surgeons for ACL reconstruction. The study was approved by the institutional research ethics committee. Informed consent was obtained at enrollment and questionnaire completion.

The study was conducted in accordance with the Declaration of Helsinki and approved by the Comité de Protection des Personnes (CPP) Île-de-France VI, Groupe Hospitalier Pitié-Salpêtrière [on 6 February 2013]. It forms part of the French prospective ACLR cohort study (ClinicalTrials.gov identifier: NCT02511158). This report follows the Transparent Reporting of Evaluations with Nonrandomized Designs (TREND) guidelines. The study was conducted using data from a cohort established in 2012, for which ethical approval was obtained in 2013. The cohort includes both a retrospective phase (data collected up to 2013) and a prospective phase (data collected thereafter). All patients included in the present analysis were recruited prospectively from 2017 onwards.

### 2.1. Study Population

One surgeon performed notchplasty in all patients operated in 2018. To create a comparable cohort with ≥2 years follow-up, patients operated in 2017 without notchplasty by the same surgeon were used as controls. Thus, a retrospective single-surgeon analysis of prospectively collected data was performed.

Inclusion criteria were patients ≥18 years, with or without meniscal injury, undergoing primary ACL reconstruction using semitendinosus and gracilis (STG) hamstring autografts. Lateral extra-articular tenodesis was performed in selected cases. Exclusion criteria: other techniques, concomitant ligament surgery, osteotomy, revision ACL, or refusal. In particular, patients requiring concomitant MCL, PCL, or posterolateral corner reconstruction were excluded. Partial sprains or isolated MCL/PCL lesions that did not require surgical treatment were included and managed non-operatively.

**Figure 1:**
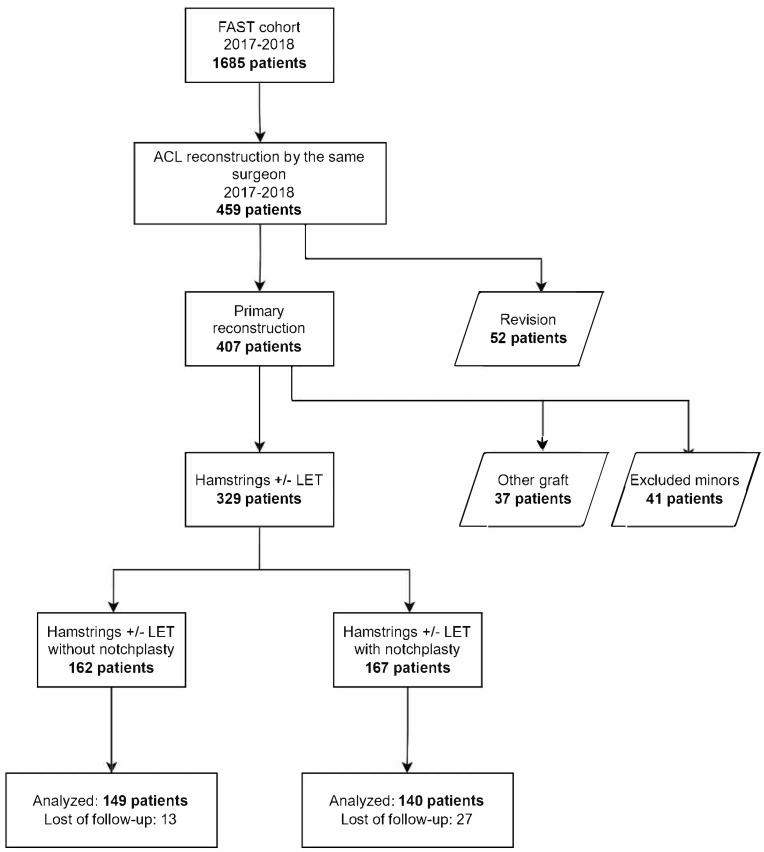
Flowchart of patient inclusion and exclusion in the study. The diagram summarizes the FAST cohort, selection by surgeon, exclusions (revision cases, minors, other grafts), and final division into notchplasty vs. non-notchplasty groups with analyzed patient numbers.

Between 2017 and 2018, 1685 patients were in the cohort; 459 underwent ACL reconstruction by the same surgeon. After excluding 52 revisions, 41 minors, and 37 with other grafts, 329 remained. Notchplasty was performed in 167 patients in 2018 (Figure 1).

Forty patients were lost to follow-up, leaving 289 (87.8%). Preoperative characteristics were comparable (Table 1), except for follow-up duration, which was longer in the 2017 non-notchplasty group (mean 35.4 months) vs. the 2018 notchplasty group (mean 27.4 months, *p* < 0.0001), explained by surgical year. Associated lesions were similar (Table 2). The population was representative of the broader ACL cohort at our institution, supporting external validity.

### 2.2. Patient Characteristics

Forty of the 329 eligible patients were lost to follow-up or declined participation, leaving 289 (87.8%) for analysis. The preoperative characteristics of the two groups were comparable (Table 1), except for the length of follow-up, which was significantly longer in the 2017 non-notchplasty group (mean 35.4 months) than in the 2018 notchplasty group (mean 27.4 months, *p* < 0.0001). Since all secondary arthrolysis procedures occurred within the first 6 months postoperatively, the difference in mean follow-up duration (35.4 vs. 27.4 months) is unlikely to have influenced the primary outcome. Both groups had a minimum follow-up of 24 months for functional outcomes.

Associated lesions were similar between groups (Table 2). The study population was representative of the broader ACL reconstruction cohort treated at our institution in terms of age, activity level, and graft type, supporting external validity

### 2.3. Study Protocol, Surgical Technique

Arthroscopic ACL reconstruction was performed with an STG graft. Grafts were prepared as quadrupled hamstring constructs unless tendon length/quality required a tripled configuration. The femoral tunnel was created via an anteromedial portal (inside-out), fixed with an Endobutton femorally and interference screw tibially.

If necessary (retear risk, pivot shift, high-level athletes), lateral extra-articular tenodesis was performed. This consisted of a modified Lemaire procedure using the iliotibial band.

In 2018, notchplasty was applied systematically as part of the operating surgeon’s standardized protocol, reflecting prevailing practice patterns at the institution at that time and aiming to minimize any potential graft–notch impingement in high-throughput clinical care.

About 3 mm of bone (width and depth, corresponding to burr size) was resected from the medial surface of the lateral condyle and notch roof with a motorized shaver to enlarge the intercondylar space and reduce graft impingement.

The postoperative protocol was standardized: immediate weight-bearing, hinged brace, and physiotherapy from week 1. Rehabilitation emphasized early quadriceps activation, hamstring relaxation, and rapid recovery of extension.

Patients were evaluated at 6 weeks and 8 months. If deficit persisted at 6 weeks, physiotherapy was intensified. At 4 months, persistent >5° deficit triggered MRI (cyclops, arthrofibrosis) [5], and, if confirmed, arthrolysis.

At 6, 12, and 24 months, patients completed online questionnaires (Websurvey.fr^®^). These included validated instruments: ACL-RSI [13], IKDC [14], Lysholm [15], KOOS [16]; plus items on return to running, pivoting, preinjury sport, time to return, and performance level. Automated reminders and follow-up phone calls were used to maximize compliance.

### 2.4. Statistical Analysis

Sample size was calculated assuming a 10% arthrolysis rate in the non-notchplasty group vs. 2% in the notchplasty group. With α = 0.05 and power = 80%, 138 per group were required.

Student’s t-test or ANOVA were used for quantitative variables; Chi-square or Fisher’s exact test for categorical variables. Analyses were performed in RStudio V1.1.463 (RStudio Inc., Boston, MA, USA).

All analyses were patient-level; no clustering adjustment was required. An intention-to-treat approach was adopted, and patients lost to follow-up were excluded without imputation.

## 3. Results

### 3.1. Rate of Arthrolysis and Other Complications

There was no significant difference in the rate of arthrolysis between the two groups: 7 patients (4.7%) in the non-notchplasty group and 7 patients (5.0%) in the notchplasty group (*p* = n.s.) (Table 3).

Among these 14 patients requiring secondary arthrolysis, intraoperative findings confirmed the presence of cyclops lesions in most cases. All arthrolysis procedures were performed within the first 6 months postoperatively, confirming that differences in mean follow-up duration between groups did not influence the primary outcome.

No additional notch reossification was observed in the notchplasty group, in contrast with the MRI evidence previously described in the literature [17].

MRI performed in these patients showed similar features in both groups, without evidence of progressive bony regrowth within the notch after notchplasty.

There was also no significant difference in the rate of retears, revisions for meniscectomy, or secondary cartilage repair.

One patient in each group developed a postoperative hematoma requiring surgical evacuation. In the notchplasty group, one patient presented with a postoperative popliteal aneurysm that was surgically treated. Additionally, one patient in the notchplasty group required surgery for resection of an intraosseous tibial tunnel cyst associated with a bioabsorbable interference screw (Table 3).

### 3.2. Functional Outcome and Return to Sport

Functional outcome data were available for 247 patients (128 in the non-notchplasty group, 119 in the notchplasty group). Forty-two patients did not complete the questionnaires despite repeated reminders and were excluded from the analysis without data imputation.

After 36 months of follow-up, there were no significant differences in functional scores (subjective IKDC, KOOS, Lysholm, Tegner, ACL-RSI) between the two groups (Table 4).

A total of 136 patients (91.3%) in the non-notchplasty group and 131 patients (93.6%) in the notchplasty group reported being satisfied or very satisfied (*p* = n.s.). Subjective instability was absent in 110 patients (73.8%) without notchplasty and in 104 patients (73.4%) with notchplasty.

There were no significant differences in the rate or timing of return to running, return to pivoting sports, or return to pre-injury sport participation. Performance levels were reported as the same or better than before the ACL tear in 109 patients (37.7%), with no significant differences between groups.

Among the 51 patients who stopped or changed sports, 12 did so for personal reasons unrelated to knee function. No association between the decision to stop sport and the presence or absence of notchplasty was observed.

## 4. Discussion

The main finding of this study was that systematic notchplasty during primary ACL reconstruction did not reduce the rate of secondary arthrolysis. Seven patients (4.7%) in the non-notchplasty group and seven patients (5.0%) in the notchplasty group required secondary arthrolysis, with no significant difference. Thus, our initial hypothesis was not confirmed.

In a series by Koga et al. [17], comparing results with and without notchplasty during double-bundle ACL reconstruction, the notchplasty group unexpectedly showed greater knee hyperextension and higher rates of secondary arthrolysis. The authors suggested that this might be explained by reossification of the notch after notchplasty. 

May et al. [18], using MRI in 33 patients, observed an average of 1.5 mm of recortication at the notchplasty site within 6 months, despite a 3 mm resection in 94% of cases. 

Similarly, Kitridis et al. [19] demonstrated that bone regrowth can occur after notchplasty over a 2-year CT follow-up. 

Kanamiya et al. [20] reported comparable MRI findings after re-notchplasty, while Murray et al. [21] identified MRI predictors of graft maturation. 

Morphological variations of the intercondylar notch have also been described by van Eck et al. [22] and Vaswani et al. [23]. 

Finally, Wang et al. [24] reported that notch morphology may change over time after ACL injury, potentially influencing impingement independently of notchplasty. 

Small notch dimensions, as described by van Eck et al. [22] and Zhang et al. [25], are recognized as risk factors for ACL injury and impingement. Fourth, extension deficits and hamstring contracture—both known contributors to cyclops syndrome [26,27]—were not specifically evaluated. Fifth, objective laxity measurements (e.g., KT-1000 ME Dmetric Corp., San Diego, USA) were not performed, so only subjective instability could be assessed. Additionally, the exclusion of patients who did not complete postoperative questionnaires may have introduced a selection bias in the analysis of functional outcomes.

Finally, the follow-up duration differed between groups (longer in 2017 vs. 2018), but since extension deficits were always diagnosed within 6 months, this likely did not bias the main outcome.

Taken together, these findings highlight that arthrofibrosis is a multifactorial problem. Factors such as graft positioning, overtensioning, extra-articular procedures, notch morphology [25], and individual healing responses all contribute, and notchplasty alone cannot be isolated as the determining variable. Nevertheless, selective notchplasty may remain justified in specific clinical scenarios, such as patients with congenitally narrow intercondylar notches or when oversized grafts are at risk of impingement. Future studies should therefore explore targeted indications rather than systematic use.

## 5. Conclusions

During single-bundle primary ACL reconstruction with hamstring autografts, systematic notchplasty did not reduce the rate of secondary arthrolysis for knee extension deficit and did not alter the overall rate of complications.

Given the underpowered design and methodological limitations, our study should be considered a pilot analysis. Larger, multicenter randomized trials with selective notchplasty indications and robust adjustment for confounders are required to draw definitive conclusions.

## Figures and Tables

**Table 1:** Preoperative demographic and clinical characteristics of patients undergoing ACL reconstruction with or without notchplasty.

	Total	Without Notchplasty	With Notchplasty	*p* Value
Number (%)	289	149 (51.6)	140 (48.4)	
Men (%)	177 (61.2)	92 (61.7)	85 (60.7)	n.s.
Age at surgery (years)	30.7	30.7	30.7	n.s.
BMI	25.3	25.5	25.2	n.s.
Practice of sport				n.s.
- sedentary, n (%)	3 (1.0)	1 (0.7)	2 (1.4)	
- occasional leisure, n (%)	25 (8.7)	17 (11.4)	8 (5.7)	
- regular leisure, n (%)	123 (42.6)	65 (43.6)	58 (41.4)	
- competitive, n (%)	122 (42.2)	58 (38.9)	64 (45.7)	
- professional, n (%)	16 (5.5)	8 (5.4)	8 (5.7)	
Type of sport				n.s.
- no sport, n (%)	2 (0.7)	0 (0.0)	2 (1.4)	
- non-pivot, n (%)	34 (11.8)	19 (12.8)	15 (10.7)	
- non-contact pivot, n (%)	53 (18.3)	31 (20.8)	22 (15.7)	
- contact pivot, n (%)	200 (69.2)	99 (66.4)	101 (72.1)	
Lachman				n.s.
- +	3 (1.0)	3 (2.0)	0 (0.0)	
- ++	204 (70.6)	97 (65.1)	107 (76.4)	
- +++	42 (14.5)	27 (18.1)	15 (10.7)	
- Unknown	40 (13.8)	22 (14.8)	18 (12.9)	
Pivot shift				n.s.
- +	156 (54.0)	68 (45.6)	88 (62.9)	
- ++	76 (26.3)	56 (37.6)	20 (14.3)	
- +++	22 (7.6)	13 (8.7)	9 (6.4)	
- Unknown	35 (12.1)	12 (8.1)	23 (16.4)	
IKDC objective				n.s.
- C	201 (69.6)	94 (63.1)	107 (76.4)	
- D	57 (19.7)	46 (30.9)	11 (7.9)	
- Unknown	31 (10.7)	9 (6.0)	22 (15.7)	
IKDC subjective	59.2	57.5	60.9	n.s.
KOOS	60	58.7	61.4	n.s.
Lysholm	70.2	69.5	71.0	n.s.
Tegner, median (range values)	7.2 (7)	7.2 (7)	7.3 (7)	n.s.
ACL-RSI	38.7	39.9	37.5	n.s.
Subjective instability				
- never, n (%)	53 (18.3)	31 (20.8)	22 (15.7)	
- during exercise, rarely, n (%)	82 (28.4)	39 (26.2)	43 (30.7)	
- during exercise, frequently, n (%)	54 (18.7)	23 (15.4)	31 (22.1)	
- occasionally, daily life, n (%)	88 (30.4)	48 (32.2)	40 (28.6)	
- constantly, n (%)	12 (4.2)	8 (5.4)	4 (2.9)	

Data are reported as means ± SD, unless otherwise mentioned. BMI: body mass index; IKDC: International Knee Documentation Committee; KOOS: Knee Injury and Osteoarthritis Outcome Score; ACL-RSI: ACL-Return to Sport after Injury. Non-pivot sport: running, cycling, swimming, golf, hiking, horseback riding, rock climbing. Non-contact pivot sport: ski, tennis, squash, badminton, volleyball, roller skating, gymnastics. Contact pivot sport: football, handball, basketball, rugby, hockey, judo, karate.

**Table 2:** Intraoperative and surgical characteristics of patients undergoing ACL reconstruction with or without notchplasty. Data are reported as mean ± SD unless otherwise specified. Meniscal lesions are detailed by management strategy (conservative, partial meniscectomy, suture). Chondral lesions are reported with type of management (conservative, debridement, microfracture). Associated ligament reconstructions (MCL, PCL, PLC) were excluded by study design; isolated sprains without reconstruction were included.

	Total	Without Notchplasty	With Notchplasty	*p* Value
Number (%)	289	149 (51.6)	140 (48.4)	
Time since 1st accident/surgery (months)	17.7	17.2	18.2	n.s.
With extralateral tenodesis	141 (48.8)	80 (53.7)	61 (43.6)	n.s.
Chondropathy, n (%)	12 (4.2)	7 (4.7)	5 (3.6)	n.s.
- conservative, n	2	1	1	
- debridement, n	6	5	1	
- microfracture, n	4	1	3	
Medial meniscal lesions, n (%)	74 (25.6)	38 (25.5)	36 (25.7)	n.s.
- conservative, n	10	7	3	
- partial meniscectomy, n	52	24	28	
- suture, n	12	7	5	
Lateral meniscal lesions, n (%)	42 (14.5)	23 (15.4)	19 (13.6)	n.s.
- conservative, n	6	4	2	
- partial meniscectomy, n	21	13	10	
- suture, n	15	6	7	
Femoral tunnel diameter (mm)	7.8	7.8	7.8	n.s.
Tibial tunnel diameter (mm)	7.8	7.8	7.8	n.s.

Data are reported as means ± SD, unless otherwise mentioned.

**Table 3:** Postoperative complications in patients undergoing ACL reconstruction with or without notchplasty. Data are expressed as number of cases and percentages. Complications include stiffness requiring arthrolysis, graft retear, secondary meniscectomy, chondral repair, postoperative hematoma, infection, tibial screw cyst, and vascular events. Contralateral ACL tears are also reported.

	Total	Without Notchplasty	With Notchplasty	*p* Value
Number of patients (%)	289	149 (51.6)	140 (48.4)	
Stiffness with arthrolysis, n (%)	13 (4.5)	6 (4.0)	7 (5.0)	n.s.
Retear, n (%)	3 (1.0)	2 (1.3)	1 (0.7)	n.s.
Post-reconstruction meniscectomy, n (%)	5 (1.7)	3 (2.0)	2 (1.4)	n.s.
Post-reconstruction chondral repair, n (%)	3 (1.0)	2 (1.3)	1 (0.7)	n.s.
Other complications	4 (1.4)	2 (1.3)	2 (1.4)	n.s.
- Postoperative hematoma with revision, n (%)	2	1	1	
- Infection, n (%)	0	0	0	
- Tibial screw cyst, n (%)	1	0	1	
- Popliteal artery aneurysm	1	1	0	
- Contralateral tear, n (%)	5 (1.7)	5 (3.4)	0 (0.0)	n.s.

**Table 4:** Functional outcomes and return-to-sport data in patients undergoing ACL reconstruction with or without notchplasty.

	**Total**	**Without Notchplasty**	**With Notchplasty**	***p* Value**
Number (%)	289	128 (51.8)	119 (48.2)	
Mean follow-up (months)	31.8	35.4 (11.1)	27.4 (4.5)	<0.0001
Subjective IKDC	86.5	86.5	86.4	n.s.
KOOS	87.5	87.2	87.9	n.s.
Lysholm	90.9	90.8	90.9	n.s.
Tegner	6.4 (10)	6.5 (10)	6.3 (10)	n.s.
ACLR-RSI	70.0	70.3	69.8	n.s.
Satisfaction				n.s.
- not satisfied, n (%)	2 (0.7)	2 (1.3)	0 (0.0)	
- fairly satisfied, n (%)	20 (6.9)	11 (7.4)	9 (6.4)	
- satisfied, n (%)	86 (29.8)	50 (33.6)	36 (25.7)	
- very satisfied, n (%)	181 (62.6)	86 (57.7)	95 (67.9)	
Subjective instability				n.s.
- never, n (%)	214 (74.0)	110 (73.8)	104 (74.3)	
- during exercise, rarely, n (%)	56 (19.4)	30 (20.1)	26 (18.6)	
- during exercise, frequently, n (%)	5 (1.7)	1 (0.7)	4 (2.9)	
- occasionally, daily life, n (%)	13 (4.5)	7 (4.7)	6 (4.3)	
- constantly, n (%)	1 (0.3)	1 (0.7)	0 (0.0)	
Return to running, n (%)	217 (75.1)	114 (76.5)	103 (73.6)	n.s.
- Mean delay (months)	10.0	9.3	11	n.s.
- Return to pivot sport, n (%)	185 (64.0)	103 (69.1)	82 (58.6)	n.s.
- Mean delay (months)	14.4	13.2	15	n.s.
Return to competitive pivot sport, n (%)	93 (32.2)	50 (33.6)	43 (30.7)	n.s.
- Mean delay (months)	15.1	15.1	15	n.s.
- Return to preinjury sport, n (%)	164 (56.7)	84 (56.4)	80 (57.1)	n.s.
- Mean delay (months)	13.5	12.9	14	n.s.
Performance level				n.s.
- stop, n (%)	10 (3.5)	3 (2.0)	7 (5.0)	
- change sport, n (%)	41 (14.2)	17 (11.4)	24 (17.1)	
- lower, n (%)	129 (44.6)	70 (47.0)	59 (42.1)	
- same, n (%)	80 (27.7)	42 (28.2)	38 (27.1)	
- higher, n (%)	29 (10.0)	17 (11.4)	12 (8.6)	

Results presented as means ± SD, unless otherwise mentioned. BMI: body mass index; IKDC: International Knee Documentation Committee; KOOS: Knee Injury and Osteoarthritis Outcome Score; ACL-RSI: ACL-Return to Sport after Injury. Non-pivot sport: running, biking, swimming, golf, hiking, horseback riding, climbing. Non-contact pivot sport: ski, tennis, squash, badminton, volley ball, roller skating, gymnastics. Contact pivot sport: football, handball, basketball, rugby, hockey, judo, karate. Data represent patients with completed questionnaires at final follow-up (n = 247); 42 patients did not respond and were excluded from the analysis.

## Data Availability

The original contributions presented in this study are included in the article. Further inquiries can be directed to the corresponding author(s).
